# S1PR1 mediates Th17 cell migration from the thymus to the skin in health and disease

**DOI:** 10.3389/fimmu.2024.1473130

**Published:** 2024-09-23

**Authors:** Jonas Engesser, Huiying Wang, Sonja Kapffer, Anna Kaffke, Anett Peters, Hans-Joachim Paust, Markus Geissen, Christian F. Krebs, Ulf Panzer, Nariaki Asada

**Affiliations:** ^1^ III. Department of Medicine, University Medical Center Hamburg-Eppendorf, Hamburg, Germany; ^2^ Hamburg Center for Translational Immunology (HCTI), University Medical Center Hamburg Eppendorf, Hamburg, Germany; ^3^ Clinic and Polyclinic for Vascular Medicine, University Heart and Vascular Center, University Medical Center Hamburg-Eppendorf, Hamburg, Germany

**Keywords:** Th17, S1PR1, thymus, skin, psoriasis

## Abstract

Th17 cells play crucial roles in host defense and the pathogenesis of autoimmune diseases in the skin. While their differentiation mechanisms have been extensively studied, the origin of skin Th17 cells remains unclear. In this study, we analyzed single-cell RNA-sequencing data and identify the presence of Th17 cells in the human thymus. Thymic Th17 cells were characterized by high expression levels of Sphingosine-1-Phosphate Receptor 1 (S1PR1), a receptor crucial for T cell egress from lymphoid tissues. In mice, Th17 cell-specific knockout of *S1pr1* resulted in the accumulation of Th17 cells in the thymus and a corresponding decrease in their numbers in the skin. Th17 cells that accumulated in the thymus exhibited a lower IL-17A production capacity compared to those in the skin, indicating that the local environment in the skin is important for maintaining the Th17 cell phenotype. Additionally, using a murine psoriasis model, we demonstrated that Th17 cell-specific knockout of *S1pr1* reduced their migration to the inflamed skin, thereby ameliorating disease progression. Collectively, our data suggest that S1PR1 mediates Th17 cell migration from the thymus to the skin, thereby modulating their functional engagement in both homeostatic and inflammatory conditions.

## 1 Introduction

T cells play key roles in adaptive immunity. They undergo a differentiation process from CD4 and CD8 double-negative precursors through a double-positive stage, ultimately emerging as single-positive CD4 or CD8 naïve T cells within the thymus (1–3). Subsequently, these cells migrate to secondary lymphoid organs or peripheral tissues, where they undergo further polarization into specific subsets, including Th17 cells (4). Predominantly located in barrier organs such as the intestine and skin, Th17 cells are pivotal in defending against pathogenic invasion by producing type 3 cytokines, namely IL-17 and IL-22, which facilitate the recruitment of immune cells to enhance pathogen clearance (5–7). While essential to host defense mechanisms, Th17 cells also contribute to the pathology of autoimmune diseases, including psoriasis (8, 9).

Psoriasis, an autoimmune condition of the skin, affects over 100 million individuals worldwide (10). Its pathogenesis is multifactorial, involving genetic predispositions, environmental triggers such as infections and medications, and lifestyle factors (11). Histologically, psoriasis is marked by epidermal thickening and the infiltration of T cells into both the dermal and epidermal layers (12). For many years, Th1 cells were considered the key drivers of psoriatic lesions due to the presence of IFN-γ-producing CD4^+^ T cells in the affected areas. However, with the identification of Th17 cells, these cells have become central to our current understanding of immune pathological processes in psoriasis. Notably, the IL-23/Th17 axis has been identified as a crucial driver of disease progression (13, 14). Therapeutic interventions targeting IL-23 (15), a cytokine essential for the maintenance of Th17 cells, as well as anti-IL-17A directed antibodies, are successfully used for the treatment of psoriasis (16–18). Nonetheless, the mechanisms underlying Th17 cell development and migration to the skin under both physiological and pathological conditions remain unclear.

In this study, we analyzed single-cell RNA sequencing (scRNA-seq) data from the human thymus and demonstrated the presence of thymic Th17 cells with high-level expression of Sphingosine-1-Phosphate Receptor 1 (S1PR1), a receptor necessary for T cell egress from lymphoid organs (19–21). Th17 cell-specific knockout of *S1pr1* resulted in the accumulation of Th17 cells in the thymus and a reduction of Th17 cells in the skin. Furthermore, using a psoriasis disease model, we showed that *S1pr1* knockout in Th17 cells reduced their migration to the skin, ameliorating disease progression. Collectively, our study identifies S1PR1 as a crucial receptor for Th17 cell migration from the thymus to the skin in both health and disease.

## 2 Materials and methods

### 2.1 Animals

*Il17a* ^Cre^ and *ROSA26* eYFP were kindly provided by Britta Stockinger (The Francis Crick Institute, UK); *S1P_1_* ^loxP^ (RRID: IMSR_JAX:019141) were kindly provided from the Cardiovascular Research Center (CVRC) UKE Hamburg. *Il17a* ^Cre^ mice were described and characterized previously (22). Moreover, we successfully created *Il17a* ^Cre^ x *ROSA26* ^eYFP^ x *S1P_1_* ^loxP^ mice (in cooperation with the transgenic core facility of the University Medical Center Hamburg-Eppendorf). All mice were on the C57BL/6J background. For cre-mediated recombination, we generated *Il17a* ^Cre/+^ heterozygous mice (*Il17a* ^Cre/+^ x *ROSA26* ^eYFP^ and *Il17a* ^Cre/+^ x *ROSA26* ^eYFP^x *S1P_1_* ^loxP^), so that IL-17A production was maintained. In all experiments, we used littermate controls (*Il17a* ^Cre/+^ x *ROSA26* ^eYFP^x *S1P_1_* ^loxP/loxP^ and *Il17a* ^Cre/+^ x *ROSA26* ^eYFP^x *S1P_1_* ^wt/wt^). All mice were raised under specific pathogen-free conditions. All animal experiments were performed according to national and institutional animal care and ethical guidelines and were approved by the local authorities.

### 2.2 Psoriasis disease model

For the induction of imiquimod (IMQ)-induced, psoriasis-like skin inflammation, one ear from 8- to 12-week-old male mice was treated daily with 5 mg IMQ cream (5% IMQ; MEDA Pharma, Bad Homburg, Germany). Control groups received vaseline applications. Thickness of ears was measured daily with a caliper. Based on this measurement, thickness score was calculated with: 0% - 20%: 0, 20% - 35%: 1, 35% - 50%: 2, 50% - 65%: 3, > 65%: 4. In addition, ears were daily scored for erythema and desquamation. Both parameters were independently scored with: 0: no, 1: mild, 2: moderate, 3: strong and 4: severe symptoms. The psoriasis score represents the mean of all three scores. H&E Staining was performed using routine laboratory methods.

### 2.3 Real-time RT-PCR analyses

Total RNA of the skin (ear) was prepared according to standard laboratory methods. Real-time PCR was performed for 40 cycles on a StepOnePlus Real-Time PCR system (Applied Biosystems, Foster City, CA) as previously described (23). All samples were run in duplicate and normalized to 18S rRNA. The following TaqMan probes (Thermo Fischer) were used: *Il17a* (Mm00439618), *S1pr1* (Mm00514644) and *18S rRNA* (Mm03928990).

### 2.4 Isolation of leukocytes from murine tissues

The leukocytes from murine thymus, lung, lymph nodes, spleen, kidneys, skin, and gut were isolated as previously described (23). Briefly, the various tissues were digested for up to 45 minutes at 37°C by adding 0.4 mg/ml collagenase D (Roche, Mannheim, Germany) and 0.01 mg/mL DNase I (Roche) to RPMI 1640 medium (Life Technologies, Karlsruhe, Germany) supplemented with 10% heat-inactivated FCS (Gibco, Eggenstein, Germany). Subsequently, the tissue was finely minced using the gentleMACS Dissociator (Miltenyi Biotec, Teterow, Germany). Single-cell suspensions were separated using Percoll density gradient centrifugation.

### 2.5 Flow cytometry

Cells were stained with fluorochrome-labeled antibodies directed against CD45, CD3, CD4, CD8, γδTCR, IL-17A, IFN-γ, CD11b, and Ly6G (Biolegend, BD Biosciences, eBioscience, or R&D Systems), as previously described (24). Flow cytometry measurements were performed using the BD FACS LSR II. Data were analyzed by using the FlowJo software (Tree Star).

### 2.6 scRNA-seq and spatial transcriptome data analysis

The Seurat package (version 5.1.0) was used for unsupervised clustering. For normalization, scaling, and principal component analysis, default parameters were used. Uniform Manifold Approximation and Projection (UMAP) was used to visualize clustering results. Differentially expressed genes in each cluster were calculated by the FindMarkers function (min.pct = 0.1), which included Wilcoxon rank sum tests. To calculate the Th17 score in scRNA-seq data, Seurat function AddModuleScore was used. Thymus scRNA-seq data were obtained from E-MTAB-8581 (only postnatal datasets were used) (https://developmental.cellatlas.io). The spatial transcriptome datasets of the healthy and psoriasis skin were obtained from https://zenodo.org/record/7562864.

### 2.7 Statistical analysis

The results are shown as the mean ± SEM when presented as a bar graph, or as single data points with the mean in a scatter dot plot. Differences between two individual experimental groups were compared using a two-tailed t test. When three or more groups were analyzed, ANOVA was used. p<0.05 was considered statistically significant.

## 3 Results

### 3.1 The thymus harbors Th17 cells expressing high levels of S1PR1

**Figure 1:**
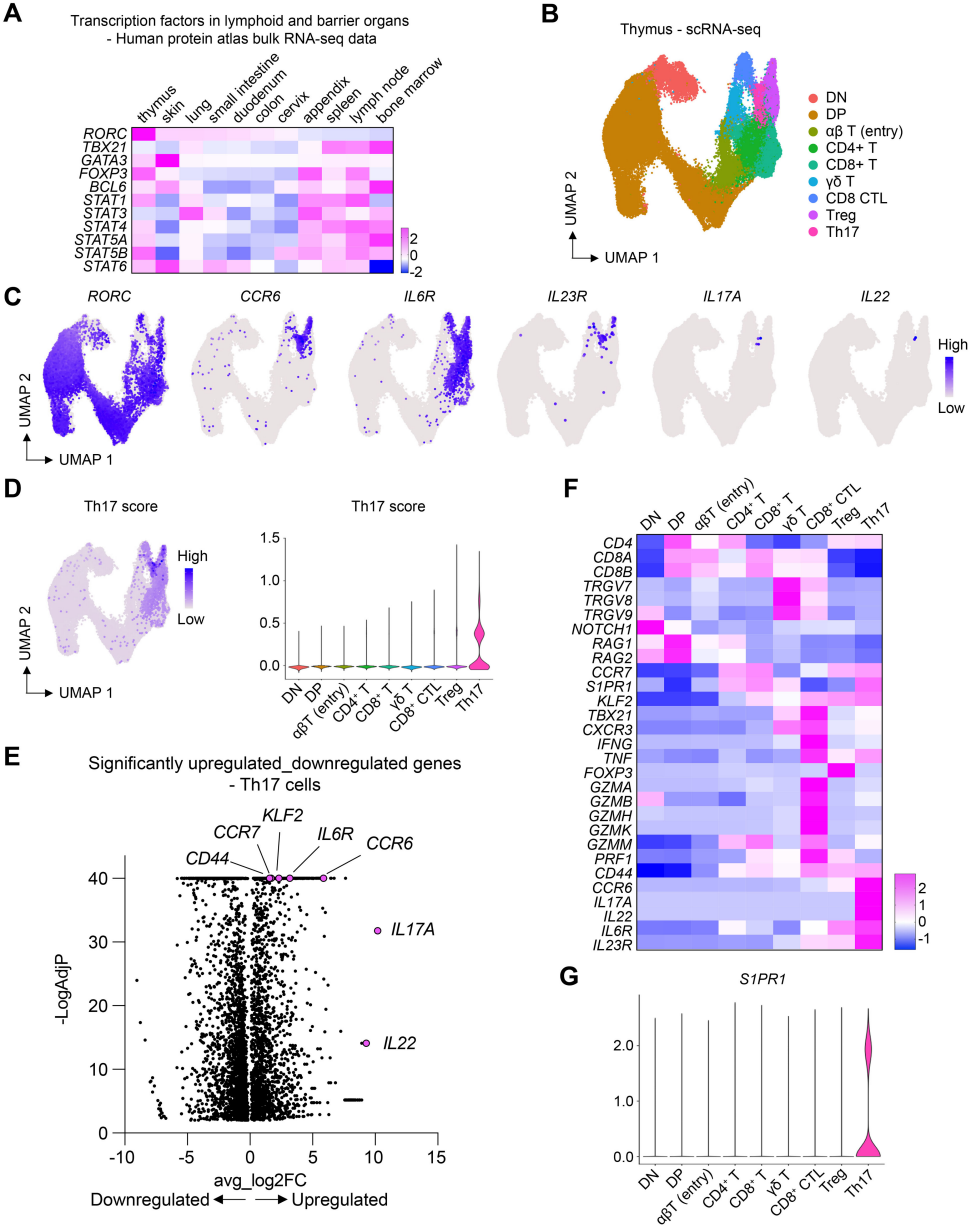
Th17 cells in the thymus express high levels of S1PR1. **(A)** Heatmap showing the expression of key transcription factors in lymphoid and barrier organs. **(B)** scRNA-seq of human thymic T cells. UMAP plot shows different T cell clusters in the thymus. **(C)** UMAP plots showing the expression of Th17 cell-associated genes. **(D)** UMAP and violin plots showing the expression of Th17 score in different cell clusters. Th17 score was calculated based on the genes shown in **(C)**. **(E)** Volcano plot showing only the differentially expressed genes in the Th17 cell cluster. **(F)** Heatmap showing the expression of marker genes. **(G)** Violin plot showing the expression of *S1PR1* in different thymic T cell clusters.

To explore the development of T cells, including Th17 cells, in lymphoid and barrier organs, we analyzed bulk RNA-sequencing data from the Human Protein Atlas (www.proteinatlas.org) (25). Analysis of key transcription factors in T cell differentiation revealed that *RORC*, which encodes the transcription factors RORγ and RORγt – crucial for T cell survival in the thymus and the development of Th17 cells, respectively (26–28) – exhibited the highest expression levels in the thymus (**Figure 1A**). The expression of *RORC* was also observed in barrier organs, including the skin. The predominant expression of *RORC* in the thymus prompted us to investigate the potential differentiation of T cells into Th17 cells within this organ.

To this end, we examined publicly available single-cell RNA-sequencing (scRNA-seq) data of thymic T cells (29). This dataset encompassed various maturation stages, including double-negative and double-positive cells, alongside more mature T cell subsets (**Figure 1B**). Notably, *RORC* expression was detectable across most thymic T cells, corroborating its established role in thymic T cell viability (**Figure 1C**). Within the mature T cells, we identified a Th17 cell cluster, characterized by the expression of markers such as *CCR6*, *IL6R*, *IL23R*, *IL17A*, and *IL22* (**Figures 1C–F**). Remarkably, these Th17 cells showed the highest levels of the receptor *S1PR1* (**Figure 1G**), pivotal for the egress of lymphocytes from the thymus and secondary lymphoid organs into the bloodstream and peripheral organs (21). The pronounced expression of *S1PR1* in thymic Th17 cells suggested their readiness for migration towards peripheral organs.

### 3.2 IL-17A fate reporter mice reveal the presence of Th17 cells in various organs

**Figure 2:**
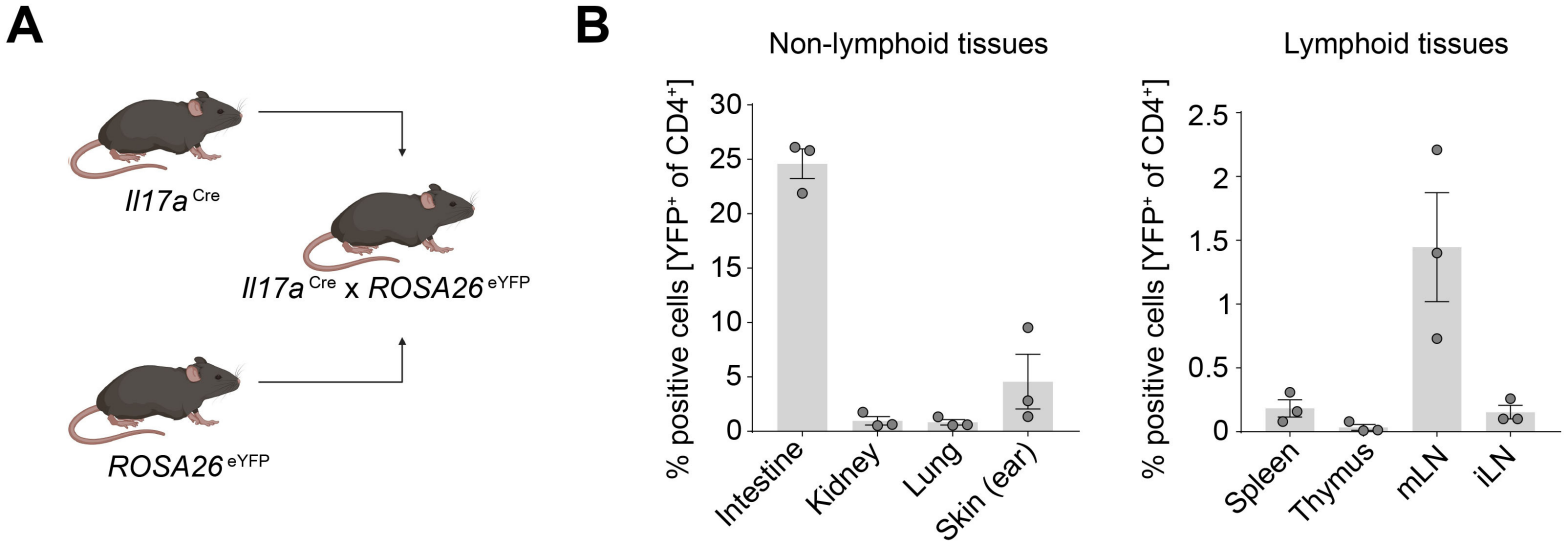
Th17 cells are enriched in the intestine and skin under homeostatic conditions. **(A)** Schematic of generation of the IL-17A fate reporter mouse. **(B)** Quantification of CD4^+^ YFP^+^ cells in lymphoid and non-lymphoid tissues under homeostatic conditions. mLN, mesenteric lymph node; iLN, inguinal lymph node.

To delineate the distribution and functional capacity of Th17 cells across various organs, including the thymus and barrier organs, we employed a fate reporter system utilizing *Il17a* ^Cre/+^ x *Rosa26* ^eYFP^ transgenic mice (**Figure 2A**). In this genetically engineered model, T cells expressing *Il17a* are irreversibly marked with enhanced yellow fluorescent protein (eYFP), enabling the precise identification of T cells which underwent Th17 cell differentiation. Consistent with our prior observations in **Figure 1**, CD4^+^ YFP^+^ T cells were detected not only in the expected barrier sites such as the skin, intestine, and mesenteric lymph nodes (mLN), where they showed the highest abundance, but also within the thymus, albeit at lower frequencies (**Figure 2B** and **Supplementary Figure 1**).

### 3.3 Th17 cell-specific *S1pr1* knockout leads to the accumulation of the cells in the thymus and reduction in the skin

**Figure 3:**
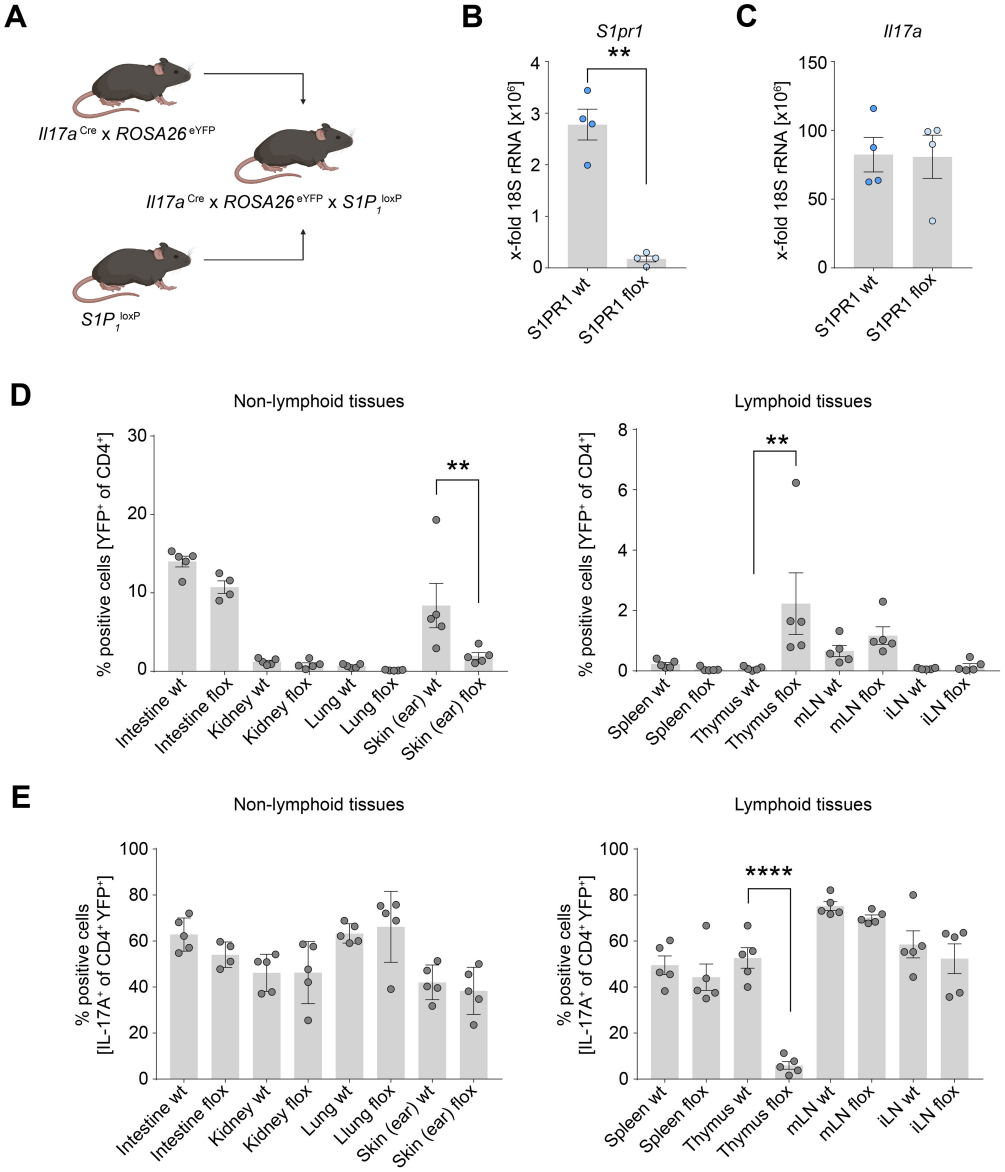
Th17 cell-specific knockout of *S1pr1* reduces Th17 cell number in the skin. **(A)** Schematic of the generation of the Th17 cell-specific *S1pr1* cKO mice. **(B)** Quantification of *S1pr1* expression levels in control and *S1pr1* knockout CD4^+^ YFP^+^ T cells. **(C)** Quantification of *Il17a* mRNA expression levels in control and *S1pr1* knockout CD4^+^ YFP^+^ T cells. **(D)** Flow cytometry analysis of IL-17A fate reporter (YFP)-positive cells in lymphoid and non-lymphoid tissues under homeostatic conditions. **(E)** Flow cytometry analysis of IL-17A-producing cells in CD4^+^ YFP^+^ cells. wt, *Il17a* ^Cre/+^ x *ROSA26* ^eYFP^x *S1P_1_* ^wt/wt^; flox, *Il17a* ^Cre/+^ x *ROSA26* ^eYFP^x *S1P_1_* ^loxP/loxP^; mLN, mesenteric lymph node; iLN, inguinal lymph node. (** P < 0.01, **** P < 0.0001).

Prompted by our observation of the highest expression of *S1PR1* in Th17 cells in the human thymus, we sought to investigate the receptor’s role in mediating Th17 cell migration to peripheral organs. To this end, we utilized *I17a* ^Cre/+^ x *Rosa26* ^eYFP^ x *S1P_1_* ^loxP/loxP^ mice (hereafter, *S1pr1* conditional knockout (cKO) mice) (**Figure 3A**). In these mice, cells expressing *Il17a* are permanently marked as eYFP^+^, while concurrently losing *S1pr1* expression, allowing us to specifically examine the impact of S1PR1 deficiency on Th17 cells. For a control group, we used *I17a* ^Cre/+^ x *Rosa26* ^eYFP^ x *S1P_1_* ^wt/wt^ mice. We observed a marked reduction in *S1pr1* expression in the *S1pr1* cKO mice CD4^+^ YFP^+^ T cells compared to the control group, without affecting *Il17a* expression levels (**Figures 3B, C**). Intriguingly, the frequency of CD4^+^ YFP^+^ T cells was significantly elevated in the thymus of the *S1pr1* cKO mice (**Figure 3D**), suggesting that Th17 cells were unable to egress from the thymus. While the prevalence of CD4^+^ YFP^+^ cells in other organs remained unchanged, a notable decline was observed in the skin (**Figure 3D**).

We further examined whether Th17 cells accumulated in the thymus retained their ability to produce IL-17A. In the control group, approximately half of the CD4^+^ YFP^+^ T cells produced IL-17A. However, the *S1pr1* cKO mice displayed a significantly reduced proportion of IL-17A production in CD4^+^ YFP^+^ T cells (**Figure 3E**), suggesting a shift away from the Th17 phenotype while they were retained in the thymus. In contrast, the capacity for IL-17A production in the skin and other organs remained unchanged between the control and *S1pr1* cKO mice (**Figure 3E**). Collectively, these findings suggest the role of S1PR1 in facilitating Th17 cell migration from the thymus to the skin under physiological conditions.

### 3.4 Human skin expresses high levels of SPHK1, an enzyme producing S1P

**Figure 4:**
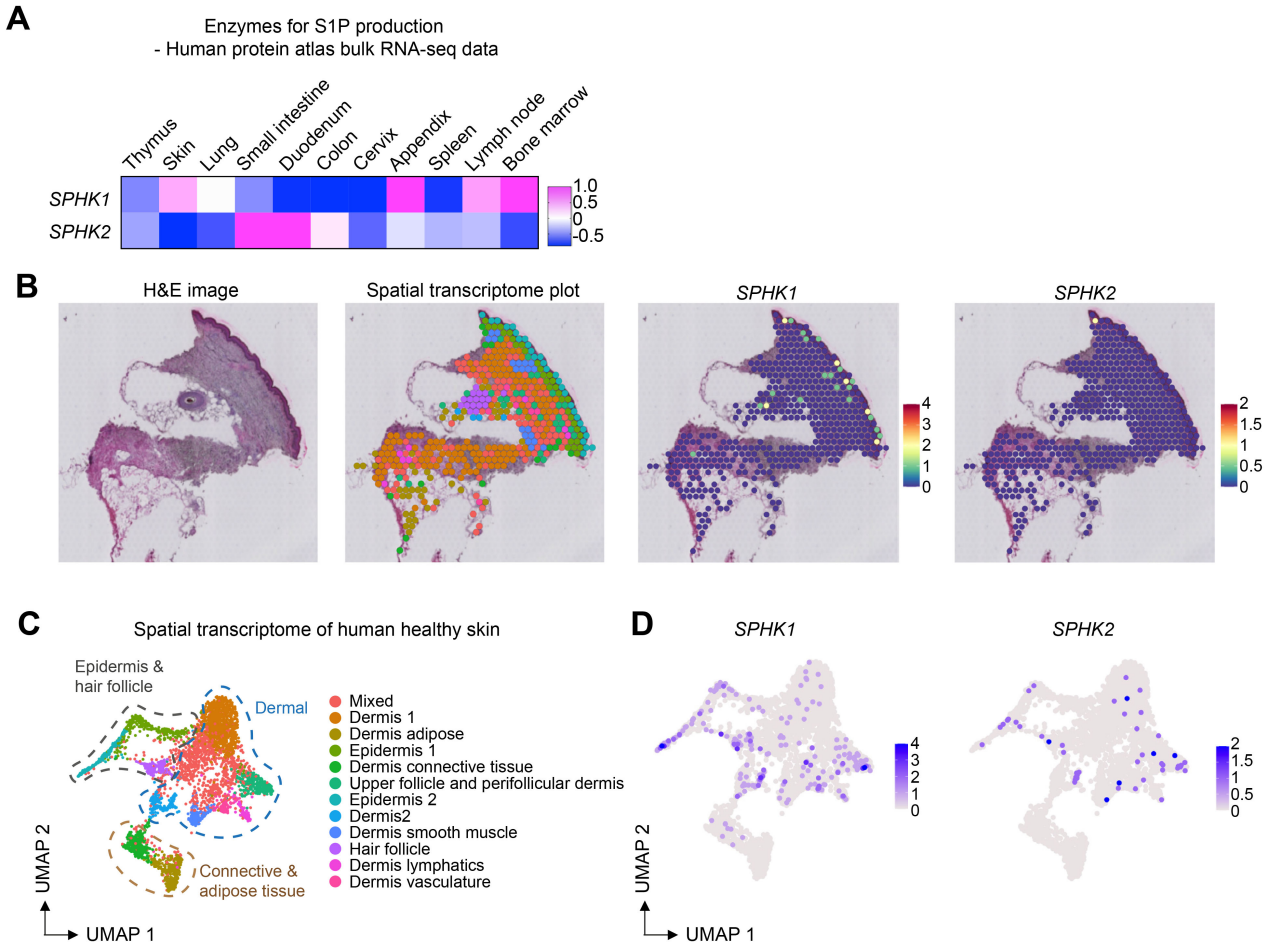
*SPHK1* and *SPHK2* expression in human healthy skin. **(A)** Heatmap showing the expression of *SPHK1* and *SPHK2* in human organs. **(B)** Representative images of HE staining, spatial transcriptome analysis of human healthy skin, and the expression of *SPHK1* and *SPHK2* in the skin. **(C)** UMAP plot of spatial transcriptomics data of human healthy skin. **(D)** UMAP plots showing the expression of *SPHK1* and *SPHK2* in human healthy skin.

S1PR1-expressing T cells are recruited by S1P, a ligand for S1PR1. S1P is produced by SPHK1 and SPHK2, which are key enzymes responsible for converting sphingosine to sphingosine-1-phosphate (S1P). These enzymes have redundant functions; therefore, the expression of either enzyme is sufficient for producing S1P and recruiting T cells. To investigate the expression of these enzymes in the skin, we examined gene expression data from the Human Protein Atlas. Notably, *SPHK1* was highly expressed in the skin as well as in the appendix, lymph nodes, and bone marrow (**Figure 4A**). The expression of *SPHK2* was mainly observed in the intestine. To further analyze the expression of these enzymes in different tissue compartments, we analyzed a publicly available spatial transcriptomics dataset of human skin. Both *SPHK1* and *SPHK2* were expressed in the epidermal and dermal compartments (**Figures 4B–D**). Once again, *SPHK1* showed more dominant expression than *SPHK2* in healthy skin.

### 3.5 Skin inflammation increases the expression levels of *SPHK1* and *SPHK2*

**Figure 5:**
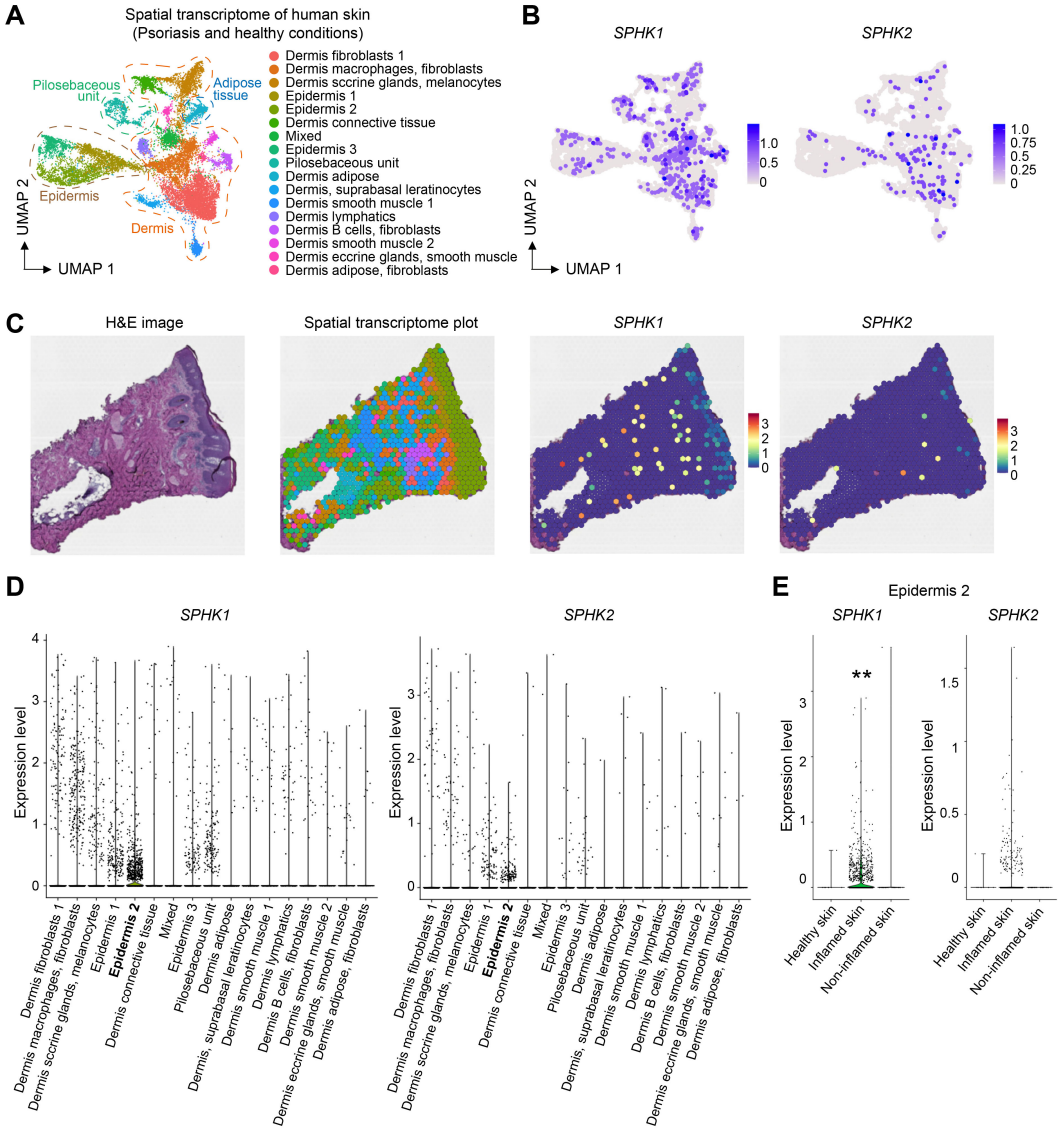
Skin inflammation increases the expression of *SHPK1* and *SPHK2*. **(A)** UMAP of spatial transcriptome analysis of human skin. **(B)** *SPHK1* and *SPHK2* expression in the dermal and epidermal layers. **(C)** Representative spatial transcriptome images of psoriatic skin and the expression of *SPHK1* and *SPHK2*. **(D)** Quantification of *SPHK1* and *SPHK2* expression levels in different tissue compartments. **(E)** Quantification of *SPHK1* and *SPHK2* expression in the epidermis 2 compartment in healthy, inflamed, and non-inflamed skin showing increased expression levels of *SPHK1* and *SPHK2* in the inflamed skin. (** P < 0.01).

Th17 cells play key roles in the skin not only in homeostasis but also in autoimmune diseases. In particular, the pathogenic involvement of Th17 cells in psoriasis is well-documented (30–32). Moreover, elevated levels of S1P have been reported in the blood of patients, particularly those with severe forms of the disease (33, 34). We therefore investigated whether the expression of S1P-producing enzymes in the skin increases in psoriasis. By analyzing spatial transcriptome data of healthy control skin and inflamed and non-inflamed skin from psoriasis patients, we found predominant expression of *SPHK1* in both the epidermal and dermal compartments (**Figures 5A–C**). Quantification of *SPHK1* and *SPHK2* expression levels across 17 compartments showed the highest expression in the epidermis 2 compartment compared to other compartments (**Figure 5D**). Notably, the expression of *SPHK1* and *SPHK2* increased in the inflamed psoriatic skin compared to healthy control skin and non-inflamed skin from psoriasis patients (**Figure 5E**). These observations suggest that Th17 cells might be more efficiently recruited to the skin during inflammation.

### 3.6 Th17 cell-specific *S1pr1* knockout ameliorates disease progression in a murine psoriasis model

**Figure 6:**
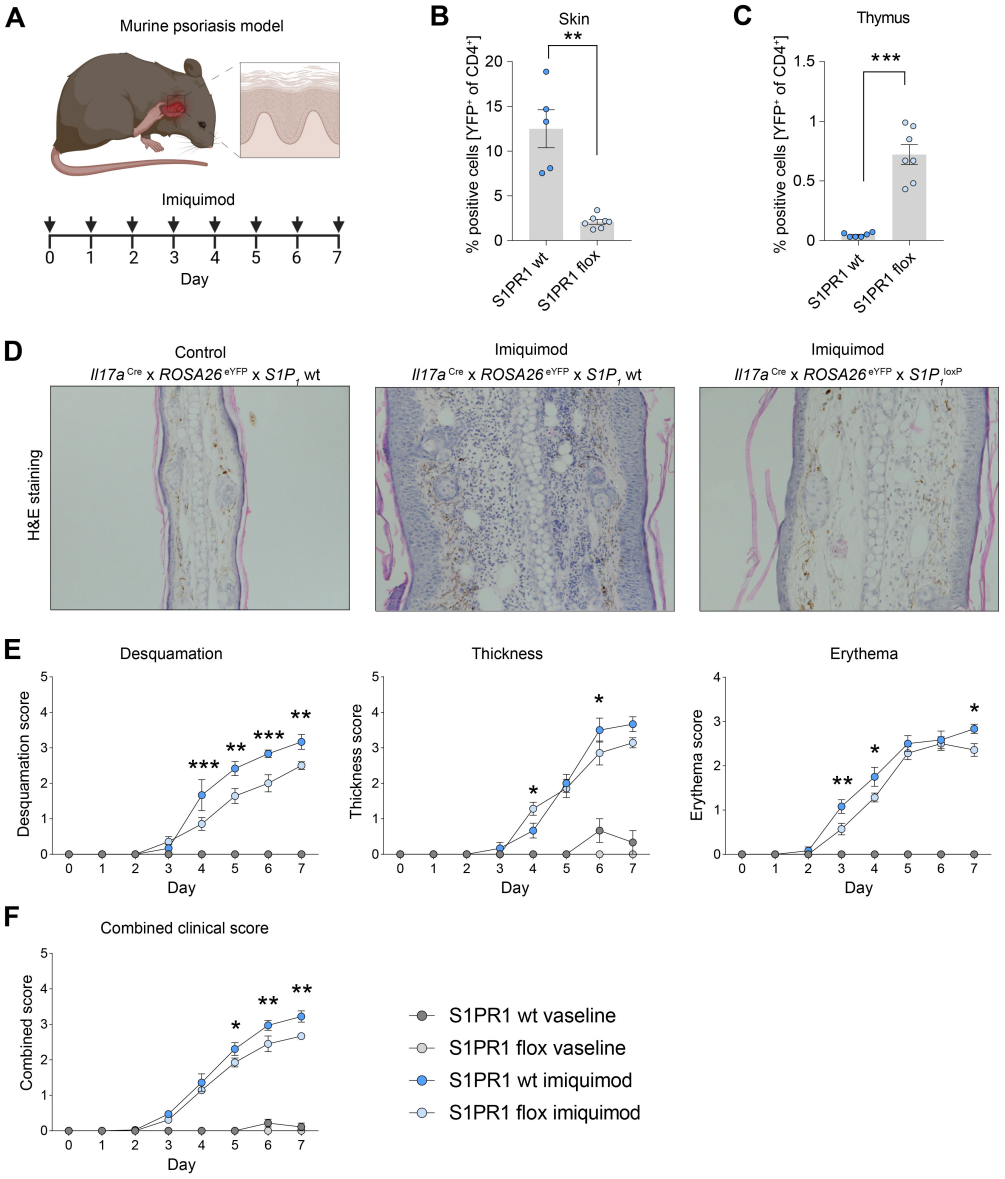
Cell specific knockout of *S1pr1* in Th17 cells ameliorates disease course in IMQ-induced psoriasis-like skin inflammation. **(A)** Schematic of the experimental set up for induction of imiquimod-induced psoriasis model. For a healthy control group, we used vaseline instead of imiquimod. **(B, C)** Flow cytometry-based quantification of IL-17A fate reporter (YFP)-positive cells in the skin and thymus under inflammatory conditions. **(D)** Representative H&E staining of control and psoriatic lesions. **(E)** Quantification of psoriatic disease severity by desquamation score, thickness score and erythema score, showing an amelioration of the disease course in Th17 cell-specific *S1pr1* cKO mice. **(F)** Combined clinical score showing a reduced severity in Th17 cell-specific *S1pr1* cKO mice. wt, *Il17a* ^Cre/+^ x *ROSA26* ^eYFP^x *S1P_1_* ^wt/wt^; flox, *Il17a* ^Cre/+^ x *ROSA26* ^eYFP^x *S1P_1_* ^loxP/loxP^.(* P < 0.05, ** P < 0.01, *** P < 0.001).

To investigate the role of S1PR1-mediated Th17 cell migration to inflamed skin, we utilized an imiquimod (IMQ)-induced murine psoriasis model and assessed the impact of Th17 cell-specific *S1pr1* knockout on disease progression (**Figure 6A**). Remarkably, in *S1pr1* cKO mice, we observed a significant reduction in Th17 cells within the skin and an increase in the number of Th17 cells in the thymus (**Figures 6B, C**), indicating that S1PR1 plays a key role in Th17 cell trafficking to inflamed skin. Furthermore, the *S1pr1* cKO mice exhibited a markedly less severe psoriatic phenotype, as evidenced by histopathological analysis (**Figure 6D**) and reductions in skin desquamation, thickness, erythema, and combined clinical scores compared to controls (**Figures 6E, F**). Taken together, these findings highlight the critical role of S1PR1 in Th17 cell migration during skin inflammation and psoriasis development.

## 4 Discussion

Th17 cells have been identified as key players not only in host defense but also in autoimmune diseases, including psoriasis. Recent advancements in omics technologies have significantly enhanced our understanding of Th17 cells (8, 31, 32). However, the developmental origin and subsequent migration mechanisms of Th17 cells are still not fully understood. Our study provides novel insights into the role of S1PR1 in the migration of Th17 cells from the thymus to the skin under homeostatic conditions. Furthermore, we demonstrate that S1PR1 mediates Th17 cell migration to inflamed skin in psoriasis, contributing to disease progression.

The conventional model of Th17 cell development begins with the migration of naïve CD4 T cells from the thymus to peripheral tissues or secondary lymphoid organs, where they differentiate into Th17 cells in the presence of cytokines such as TGF-β, IL-6, and IL-23 (35, 36). However, the presence of IL-17A-producing T cells in the thymus has also been reported (37, 38).

Our scRNA-seq data analysis identified a distinct population of thymic Th17 cells with high expression of *S1PR1* (**Figure 1G**). Given the role of S1PR1 in cell migration, we aimed to identify its role in Th17 cell trafficking from the thymus to peripheral organs. In the analysis of Th17 cell-specific *S1pr1* cKO mice, we found that Th17 cells accumulated in the thymus. Notably, among the peripheral tissues of the cKO mice, the skin showed the most striking reduction in the number of Th17 cells (**Figure 3D**). These data indicate that S1PR1 mediates the trafficking of thymic Th17 cells to the skin under homeostatic conditions, providing new insights into the origin of skin Th17 cells.

The recruitment of S1PR1-expressing T cells requires SPHK1 or SPHK2, the enzymes responsible for S1P production (39). Following synthesis by SPHK1 or SPHK2 with in cells (40–42), S1P is transported out of cells *via* specific S1P transporters (43), forming an S1P gradient that guides the migration of T cells. The role of SPHK2 in Th17 cell development in the skin has been previously reported (44, 45). However, given our observation of high levels of *SPHK1* (but not *SPHK2*) in the skin (**Figure 4A**), we speculate that SPHK1 is the dominant enzyme contributing to S1P production in the skin, thereby recruiting S1PR1-expressing Th17 cells.

The importance of S1P-S1PR1 signaling in psoriasis has been well-documented. High levels of S1P have been reported in the blood of psoriasis patients, particularly in severe cases (33, 34). Furthermore, the S1PR1 antagonist ponesimod has demonstrated clinical efficacy in a phase 2 trial, significantly ameliorating psoriatic symptoms (46). Despite these findings in clinical settings, the mode of action and molecular mechanism of S1P-S1PR1 signaling in psoriasis remain unclear. S1P has been reported to increase the expression of proinflammatory cytokines such as TNF-α in human keratinocytes *in vitro* (47). On the other hand, another study showed that S1P inhibits the proliferation of keratinocytes and induces their differentiation (48, 49), which might be beneficial for psoriasis patients. Therefore, the role of S1P-S1PR1 signaling in keratinocytes in psoriasis is complex and requires further analysis in *in vivo* experiments. In the immune system, S1PR1 is known to be important for T cell egress, leading to the speculation that blocking S1PR1 in psoriasis might show beneficial effects by blocking T cell egress from lymphoid organs. However, clear evidence supporting this idea has been missing. To address this, we induced a psoriasis model in Th17 cell-specific *S1pr1* cKO mice and found that knocking out S1PR1 in Th17 cells resulted in fewer Th17 cells in the skin and a milder disease course. These findings suggest that S1PR1-targeting treatment has beneficial effects in psoriasis patients, at least partly, by suppressing Th17 cell trafficking to the inflamed skin lesions.

In this study, we utilized *Il17a^Cre^* mice to investigate the role of S1PR1 in Th17 cells. However, this transgenic system has certain limitations. Since *Il17a* can be expressed not only by Th17 cells but also by other cell types, such as γδT cell (50), we cannot entirely rule out the possibility that the migration of γδT cells was also affected in our S1PR1 cKO experiments. Additionally, for transcriptome analysis, we used human datasets due to the lack of appropriate murine datasets. However, it is important to consider that there may be interspecies differences between humans and mice that could influence the results.

In summary, our study demonstrated that thymic Th17 cells express high levels of S1PR1 and migrate to healthy skin, where SPHK1 is highly expressed. In a psoriasis model, targeting S1PR1 in Th17 cells reduces their migration to the skin and ameliorates skin inflammation. These findings provide new insights into the origin of skin Th17 cells in health and disease.

## Data Availability

The original contributions presented in the study are included in the article/**Supplementary Material**. Further inquiries can be directed to the corresponding author.
