# Supplementary material for: S1PR1 mediates Th17 cell migration from the thymus to the skin in health and disease
Source: Front Immunol. 2024 Sep 23;15:1473130. doi: 10.3389/fimmu.2024.1473130 (PMC11459589; doi:10.3389/fimmu.2024.1473130)
Supplement: Supplementary file 1 [file Image1.pdf]

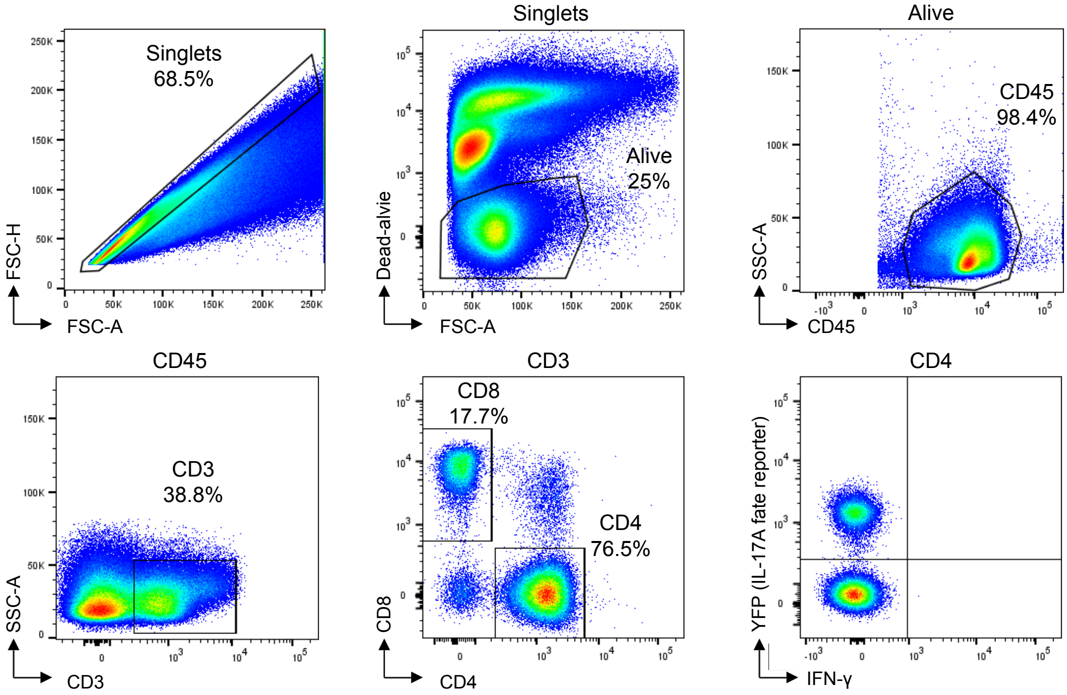

Supplementary Figure 1: Gating strategy for the identification of IL-17A fate reporter cells via flow cytometry
